# Incidence of human papillomavirus–related cancers among males and females aged 15-34 years in the United States

**DOI:** 10.1093/jncics/pkad016

**Published:** 2023-02-23

**Authors:** Fangjian Guo, Victor Adekanmbi, Christine D Hsu, Abbey B Berenson

**Affiliations:** Department of Obstetrics and Gynecology, The University of Texas Medical Branch at Galveston, Galveston, TX, USA; Center for Interdisciplinary Research in Women’s Health, The University of Texas Medical Branch at Galveston, Galveston, TX, USA; Department of Obstetrics and Gynecology, The University of Texas Medical Branch at Galveston, Galveston, TX, USA; Center for Interdisciplinary Research in Women’s Health, The University of Texas Medical Branch at Galveston, Galveston, TX, USA; Department of Obstetrics and Gynecology, The University of Texas Medical Branch at Galveston, Galveston, TX, USA; Center for Interdisciplinary Research in Women’s Health, The University of Texas Medical Branch at Galveston, Galveston, TX, USA; Department of Obstetrics and Gynecology, The University of Texas Medical Branch at Galveston, Galveston, TX, USA; Center for Interdisciplinary Research in Women’s Health, The University of Texas Medical Branch at Galveston, Galveston, TX, USA

## Abstract

Postmarket surveillance of the incidence of human papillomavirus (HPV)–related cancers is essential to monitor the effectiveness of HPV vaccines. We directly compared HPV-related cancer incidences during the pre- and postvaccine era to assess the effects of HPV vaccination among vaccine-eligible age groups in the United States using data from the US Cancer Statistics database. The 5-year average annual incidence rates for HPV-related cancers decreased in 2015-2019 compared with 2002-2006 among females aged 15-24 years and 25-34 years. Overall, a decrease in young males was not observed, whereas males aged 25-34 years experienced a slight decline in oropharyngeal squamous cell carcinoma between 2005-2009 and 2015-2019. Incidence rates for HPV-related cancers statistically significantly decreased in the vaccine era compared with the prevaccine era among females aged 15-34 years, suggesting the potential early effects of the introduction of HPV vaccination in the United States.

Human papillomavirus (HPV) vaccines have been available in the United States since 2006 to prevent HPV-related cancers, which include cancers of the cervix, vagina, vulva, penis, anus, rectum, and oropharynx (1,2). The Advisory Committee on Immunization Practices recommends routine HPV vaccination of adolescents at age 11-12 years, catch-up vaccination for all persons through age 26 years who are not adequately vaccinated, and vaccination based on shared clinical decision making for persons aged 27-45 years who are not adequately vaccinated (3,4). HPV vaccines have been recommended for females since 2006 and for males since 2009 (5).

Previous studies estimated HPV-related cancer incidence from 2012 to 2016 (1) and the association between HPV vaccination and trends in HPV-related cancers from 2001 to 2017 in the United States (6). As the sojourn time from HPV acquisition for HPV-related cancers is long, direct comparison of HPV-related cancer incidences between the prevaccine era and most recent years among vaccine-eligible age groups will provide a better evaluation of the effects of HPV vaccination in the United States. We compared the 5-year average annual incidence of HPV-related cancers in the 5 years before HPV vaccine introduction among females (2002-2006) and among males (2005-2009) aged 15-34 years and the most recent 5 years in the vaccine era (2015-2019) in the United States.

We used data from the US Cancer Statistics (USCS) 2001-2019 database, which covers almost the entire US population. We included primary cancer cases of HPV-related cancers (variable supplied by SEER*Stat 8.4.0.1), including oropharyngeal squamous cell carcinoma, anal and rectal squamous cell carcinoma, vulvar squamous cell carcinoma, vaginal squamous cell carcinoma, penile squamous cell carcinoma, and cervical carcinoma (Table 1). The study was exempt from institutional review board review at the University of Texas Medical Branch at Galveston.

**Table 1.** Age-adjusted incidence of HPV-related cancers among females aged 15-34 years in the United States during 2002-2006 and 2015-2019

Characteristics by age group	No. of cases		Incidence (per 1 000 000 person-years) (95% CI)^a^		Rate ratio vs 2002-2006
	2002-2006	2015-2019	2002-2006	2015-2019	
Age group					
15-24 y					
HPV-related cancers^b^	1163	428	11.13 (10.5 to 11.79)	3.85 (3.5 to 4.24)	0.35 (0.31 to 0.39)
Vulvar squamous cell carcinoma	314	60	3.02 (2.7 to 3.38)	0.55 (0.42 to 0.7)	0.18 (0.13 to 0.24)
Cervical carcinoma	790	343	7.54 (7.02 to 8.08)	3.08 (2.76 to 3.43)	0.41 (0.36 to 0.46)
25-34 y					
HPV-related cancers	9853	9595	101.92 (99.92 to 103.95)	88.99 (87.22 to 90.79)	0.87 (0.85 to 0.90)
Oropharyngeal squamous cell carcinoma	141	138	1.46 (1.23 to 1.73)	1.27 (1.07 to 1.51)	0.87 (0.68 to 1.11)
Anal and rectal squamous cell carcinoma	158	193	1.64 (1.39 to 1.91)	1.8 (1.56 to 2.08)	1.10 (0.89 to 1.37)
Vulvar squamous cell carcinoma	1052	627	10.89 (10.24 to 11.57)	5.84 (5.39 to 6.31)	0.54 (0.48 to 0.59)
Vaginal squamous cell carcinoma	93	67	0.96 (0.77 to 1.18)	0.63 (0.48 to 0.79)	0.65 (0.47 to 0.90)
Cervical carcinoma	8409	8570	86.97 (85.12 to 88.85)	79.45 (77.77 to 81.15)	0.91 (0.89 to 0.94)

^a^ Annual incidence rates were calculated as the number of cases per 1 000 000 persons and were age-adjusted to the 2000 US standard population. Differences in age-adjusted rates were evaluated using rate ratios (RRs) and the corresponding 95% confidence intervals (CIs). The Tiwari method was used to determine the confidence intervals. HPV = human papillomavirus.

^b^ Definitions of HPV-related cancer are as follows. All are histologically confirmed.

Oropharyngeal squamous cell carcinoma: the International Classification of Disease for Oncology, Third Edition (ICD-O-3) site codes: C01.9, C02.4, C02.8, C05.1, C05.2, C09.0, C09.1, C09.8, C09.9, C10.0-C10.4, C10.8, C10.9, C14.0, C14.2, and C14.8.

ICD-O-3 histologic codes 8050-8086 and 8120-8131.

Anal and rectal squamous cell carcinoma: ICD-O-3 site codes C20.9, C21.0-C21.2, C21.8. ICD-O-3 histologic codes 8050-8084 and 8120-8131.

Vulvar squamous cell carcinoma: ICD-O-3 site codes C51.0-C51.2, C51.8, and C51.9.

ICD-O-3 histologic codes 8050-8084 and 8120-8131. Only female patients are included.

Vaginal squamous cell carcinoma: ICD-O-3 site code C52.9.

ICD-O-3 histologic codes 8050-8084 and 8120-8131. Only female patients are included.

Cervical carcinoma: ICD-O-3 site code C53.0, C53.1, C53.8, and C53.9.

ICD-O-3 histologic codes 8010-8671 and 8940-8941. Only female patients are included.

The age-adjusted annual incidence rates for HPV-related cancers from 2001 to 2019 by sex and age group are shown in Figure 1, A-D. The 5-year average annual incidence rates for HPV-related cancers decreased in 2015-2019 compared with 2002-2006 among females aged 15-24 years (3.85 vs 11.13 per 1 000 000; rate ratio [RR] = 0.35, 95% CI = 0.31 to 0.39; Table 1) and aged 25-34 years (88.99 vs 101.92 per 1 000 000; RR = 0.87, 95% CI = 0.85 to 0.90). The incidence of oropharyngeal squamous cell carcinoma decreased in 2015-2019 compared with 2005-2009 among males aged 25-34 years (0.81 vs 1.75 per 1 000 000; RR = 0.81, 95% CI = 0.66 to 0.99; Supplementary Table 1, available online).

**Figure 1.**
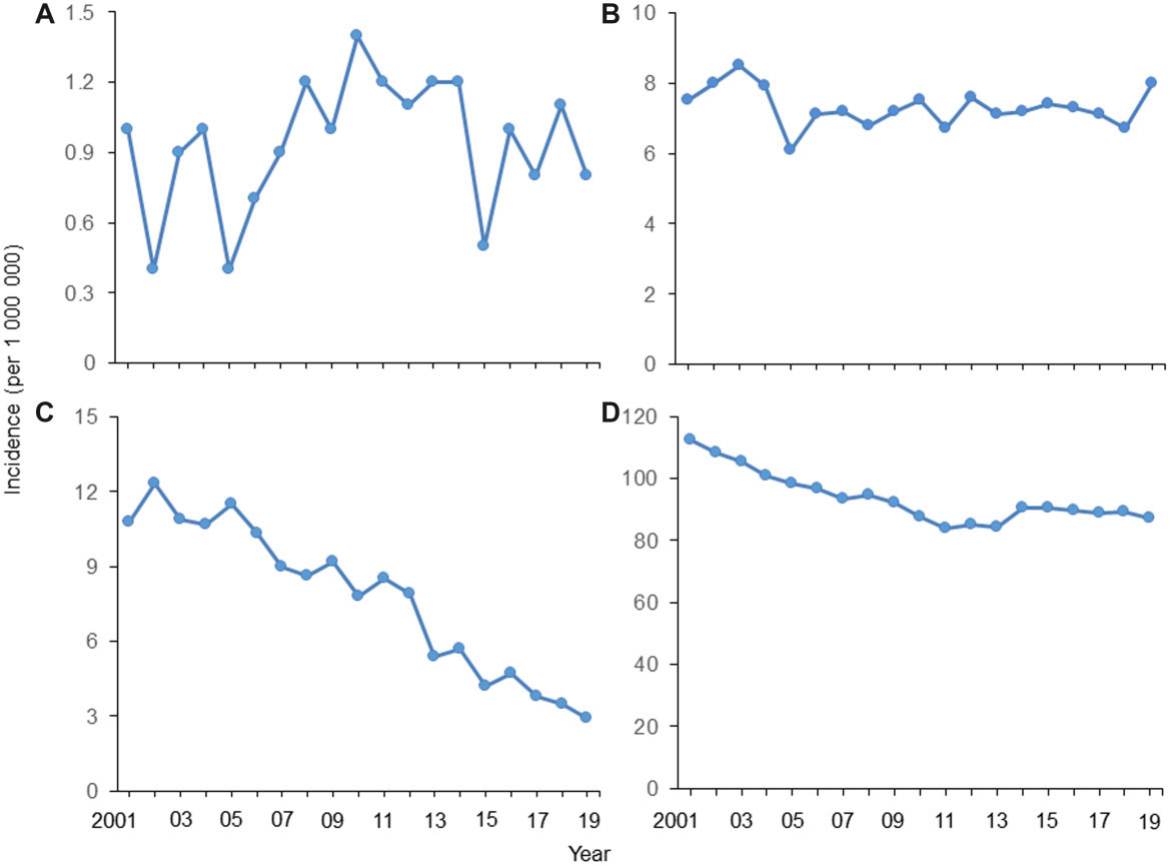
Age-adjusted incidence of HPV-related cancers from 2001 to 2019 among US males and females aged 15-34 years. **(A)** Males aged 15-24 years, **(B)** males aged 25-34 years, **(C)** females aged 15-24 years, **(D)** females aged 25-34 years.

Using data from the USCS database, we assessed the incidence of HPV-related cancers before and after the introduction of the HPV vaccine. We found that incidence rates for HPV-related cancers were lower in 2015-2019 compared with 2002-2006 among females aged 15-34 years.

Reductions in vaccine-type HPV were seen among females aged 14-19 and 20-24 years, 88% and 81% respectively, from 2015 to 2018 compared with prevaccine era rates for those age groups (7). Decreases in vaccine-type HPV prevalence have been noted not only among those who are vaccinated but also among unvaccinated populations as a result of herd immunity (8-10). Cancer screening [eg, the introduction of HPV testing in cervical cancer screening by US Preventive Services Taskforce guidelines in 2012 (11)] and declining smoking rates may also help explain the decreased incidence we observed among females. Tobacco use, a risk factor for HPV-related cancers, is decreasing in the United States (12-14). Daily smokers decreased from 17% to 11% from 2005 to 2015 (15). We also observed a decrease in incidence of HPV-related cancers in females prior to the introduction of the vaccine, potentially attributable to cancer screening and decreased smoking rates.

A decrease in HPV-related cancers among young males was not observed, likely because of low vaccination rates in this population (16) and that the main HPV-related cancer (oropharyngeal cancer) in men develops later in life (17). We observed a slight decrease in oropharyngeal squamous cell carcinoma among males aged 25-34 years, though whether this decrease is attributable to HPV vaccination is to be determined. A study using Surveillance, Epidemiology, and End Results data also projects that the current HPV vaccination rates will not cause a clinically significant reduction in oropharyngeal cancers through 2045, as older patients without HPV vaccinations are at highest risk (18). In addition, although the HPV vaccine became available in the United States in 2006 and was recommended for females, the vaccine was approved by the Food and Drug Administration for use in males in 2009, which may also contribute to a lag in benefits seen among males (5). Low HPV vaccination rates among boys could be the result of early approval of HPV vaccination for females, earlier female-centric social marketing strategies for HPV vaccination, and more frequent use of health care in females (19,20). HPV vaccination initiation and completion rates among boys aged 9-12 years gradually caught up with girls in recent years, although HPV vaccination coverage within ages 9 to 12 years is still suboptimal (21). Additional efforts are needed to improve HPV vaccination coverage and cancer screening rates, especially considering the unsatisfactory vaccine uptake among young individuals (16) and decreased screening rates among young women (22).

A strength of our study is the use of the USCS database, which covers more than 99% of the US population. This study also has limitations. The USCS database does not include information on HPV genotype status in cancer tissue. HPV-related cancers here are based on anatomic sites, and about 80% of them are caused by HPV infections (1). Important known risk factors, such as smoking, were also not available in the data. Furthermore, as the sojourn time issue affects all HPV-related cancers, longer follow-up data are needed to assess the full effect of HPV vaccination when vaccinated people get older.

Incidence rates for HPV-related cancers decreased in the vaccine era compared with the prevaccine era among females aged 15-34 years, suggesting the potential early effects of the introduction of HPV vaccination in the United States. Future studies should continue assessing trends in HPV-related cancers, especially as HPV vaccination coverage has increased across the years, with 75% of adolescents aged 13-17 years having received at least 1 dose in 2020 (23).

## Supplementary Material

pkad016_Supplementary_Data

## Data Availability

Data used in this study are United States Cancer Statistics public use databases, which are publicly available through the Centers for Disease Control and Prevention (CDC) website (https://www.cdc.gov/cancer/uscs/public-use/index.htm).
