# Supplementary material for: Incidence of human papillomavirus–related cancers among males and females aged 15-34 years in the United States
Source: JNCI Cancer Spectr. 2023 Feb 23;7(2):pkad016. doi: 10.1093/jncics/pkad016 (PMC10029840; doi:10.1093/jncics/pkad016)
Supplement: pkad016_Supplementary_Data [file pkad016_supplementary_data.pdf]

Supplementary Table 1. Age-adjusted incidence of HPV-related cancers among 15-34 year old males in the US during 2005-2009 and 2015-2019.

	No. of Cases		Incidence (per 1,000,000 person-years)		Rate ratio vs. 2005-2009
	2005-2009	2015-2019	2005-2009	2015-2019	
Age group					
15-24 years old					
HPV-related cancers	93	99	0.83(0.67-1.02)	0.85(0.69-1.04)	1.03(0.77-1.38)
Oropharyngeal squamous cell carcinoma	17	23	0.15(0.09-0.24)	0.2(0.13-0.3)	1.31(0.67-2.62)
Anal and rectal squamous cell carcinoma	31	47	0.28(0.19-0.39)	0.41(0.3-0.54)	1.47(0.91-2.39)
Penile squamous cell carcinoma	45	29	0.4(0.29-0.54)	0.25(0.17-0.36)	0.62(0.38-1.02)
25-34 years old					
HPV-related cancers	670	809	6.88(6.37-7.43)	7.31(6.81-7.83)	1.06(0.96-1.18)
Oropharyngeal squamous cell carcinoma	209	193	2.17(1.88-2.48)	1.75(1.51-2.02)	0.81(0.66-0.99)
Anal and rectal squamous cell carcinoma	245	388	2.53(2.22-2.87)	3.51(3.16-3.87)	1.39(1.18-1.63)
Penile squamous cell carcinoma	216	228	2.19(1.91-2.5)	2.05(1.79-2.33)	0.94(0.77-1.13)

Definitions of HPV-related cancer are as follows. All are histologically confirmed.

Oropharyngeal squamous cell carcinoma: the International Classification of Disease for Oncology, Third Edition (ICD-O-3) site codes C01.9, C02.4, C02.8, C05.1, C05.2, C09.0, C09.1, C09.8, C09.9, C10.0-C10.4, C10.8, C10.9, C14.0, C14.2, and C14.8.

ICD-O-3 histologic codes 8050-8086 and 8120-8131.

Anal and rectal squamous cell carcinoma: ICD-O-3 site codes C20.9, C21.0-C21.2, C21.8. ICD-O-3 histologic codes

8050-8084 and 8120-8131.

Penile squamous cell carcinoma: C60.0-C60.2, C60.8 and C60.9.

ICD-O-3 histologic codes 8050-8084 and 8120-8131. Only female patients are included.
